# "They Really Save Us From Ourselves": Autism Researchers’ Insights on Community Engagement and Ethics

**DOI:** 10.1007/s11673-025-10511-x

**Published:** 2026-05-18

**Authors:** Chloe Silverman, Michael Yudell, Amy Carroll-Scott

**Affiliations:** 1 Department of Politics and Center for Science, Technology, & Society, College of Arts and Sciences, Drexel University, Philadelphia, PA 19104 USA; 2 College of Health Solutions, Arizona State University, Phoenix, AZ 85004 USA; 3 Department of Community Health and Prevention, Dornsife School of Public Health, Drexel University, Philadelphia, PA 19104 USA

**Keywords:** Disability, Research ethics, Community engagement, Autism, Representation

## Abstract

Autism research depends on the participation of autistic children and adults as well as their families, but researchers have not routinely involved autistic people in the planning or execution of research. Community-engaged research practices, standard in other fields such as areas of public health, are gaining acceptance in autism research, although some researchers and other stakeholders remain concerned about their appropriateness, feasibility, or utility. This study, part of a larger project on stakeholder views on the ethics of community engagement practices in autism research, examines researchers’ ethical beliefs about community engagement. We find that the researchers with whom we spoke cite multiple specific ethical and epistemological benefits of these practices and relatively few risks. Barriers to community engagement are, in their view, often institutional and practical, including the longer timelines of community-engaged research, resistance or lack of comprehension on the part of funding organizations and grant reviewers, the need for additional resources to support appropriate compensation, and ensuring that community stakeholders and researchers have a shared understanding of terminology.

## Introduction and Background

The turn to community engagement and participatory research is a relatively recent and comparably limited phenomenon within the field of autism research. However, researchers have expressed enthusiasm for the idea of incorporating stakeholder priorities and values in autism research design. Prior work has established stakeholders’ desire for engagement, and groups using these practices have offered concrete ideas about how best to implement community engagement in autism studies as well as the design of autism interventions and supports (Pellicano and den Houting 2022; den Houting et al. 2021; Cascio et al. 2020; Nicolaidis et al. 2019; Fletcher-Watson et al. 2017; Pellicano, Dinsmore, and Charman 2014a, b). There are also promising examples of community-engaged autism research that demonstrate the potential for these practices to both enhance research and strengthen respect and collaboration between researchers and stakeholder groups (e.g. Donaldson et al. 2023; Benevides et al. 2020; Nicolaidis et al. 2015; Nicolaidis et al. 2012).

However, such practices are far from the norm in autism research. This is despite the fact that they are common in other areas such as community health, where robust methods for collaborating with communities have been refined and utilized for decades, or other areas of intervention, social services, and policy design for populations with other disabilities, as demonstrated by the literature on codesign (e.g. Ashburner et al 2023; Benz et al 2024; Fraser-Barbour et al. 2025). Community-engaged research practices have been particularly useful in helping researchers challenge conventional wisdom in addressing complex, multifactorial issues like “low birth weight, substance abuse, depression, teen pregnancy, asthma, and inadequate access to care” (Lasker and Weiss 2003, 18). The relative lack of this approach in autism research is surprising, given that researchers focused on autism are aware that stakeholders, including autistic people, parents and caregivers, and service providers have strong and complex opinions about the most urgent topics for research, appropriate methods for addressing those topics, and the best ways to interpret study results. These varied opinions include those of parents seeking research considering environmental causes of autism or autistic activists wary of research focused on genetic testing and diagnosis or calling for research on communication or healthcare access (e.g. Nicolaidis et al. 2015). Community engagement can offer one response to the presence of these competing values, priorities, and agendas throughout the research process.

Community-engaged research can take multiple forms, ranging from stakeholder advisory boards solicited for periodic input, to community-based participatory research (CBPR), in which community members participate throughout the research process, from initial conception of the research topic and research questions through to dissemination (Minkler and Wallerstein 2008; Fletcher-Watson et al. 2017). Such processes ensure meaningful input from those most affected by the research and can address significant problems that may arise with research feasibility or ethics before they occur. They also can serve an important function of capacity building, helping autistic individuals and family members enter research careers, or enabling them to recruit and mentor additional community partners, ensuring that the costs and benefits of participation are equitably distributed. Finally, they help to better align research programmes with community priorities. For example, findings from community surveys suggest that multiple stakeholder groups, including autistic adults, family members, practitioners, and researchers, believe that disproportionate funding support for basic research may come at the expense of investigating and developing practical strategies to educate, support, and accommodate autistic people (Pellicano, Dinsmore, and Charman 2014a). Similarly, there is growing evidence of the efficacy of codesign practices in autism supports, where technologies and programmes designed with the guidance of autistic users have greater relevance to their needs and higher degrees of accessibility.

That community engagement ought to be central to autism research is increasingly recognized, at least in principle, by researchers, though at least one study suggests that autistic adults and family members disagree with autism researchers about the degree and success of researchers’ community engagement efforts (Pellicano, Dinsmore, and Charman 2014b). This slow uptake may be because, from the perspective of researchers, community engagement is not without potential drawbacks, many of them associated with the high degree of contestation among autism stakeholder groups regarding even basic features of the condition. Simply accounting in research for the broad range of strengths, weaknesses, and support needs that characterize this diagnosis can prove a challenge (Hollin and Pearce 2019; Garfield and Yudell 2019; Pickard et al. 2021; Largent et al. 2018). Because of these competing beliefs, researchers may fear a loss of their ability to determine which problems most warrant their attention and the expenditure of research funds, decisions that can respond not only to community needs or researcher interest but also to the priorities of professional communities and funders. Researchers may also have concerns about the scientific integrity of community-engaged research processes, which can affect their professional credibility and ability to publish. Utilizing community engagement or including autistic co-researchers may challenge autism researchers’ implicit and explicit attitudes toward autism, suggesting that the choice to undertake community engagement has epistemological consequences (Botha and Cage 2022).

A major concern expressed in both journalistic coverage and academic articles is the question of how best to include community input, what forms community engagement ought to take, and how to identify and define relevant communities for the purpose of engagement. McCoy (2020) and colleagues identify this as a problem of “partial representation” when it occurs in the context of autism policy recommendations. They argue that some stakeholder communities, for example, autistic adults with few support needs, are treated as representative of autism stakeholders as a whole, leaving out the needs and interests of, for example, children with significant support needs and their families. A distinct though related concern is the degree to which advancing an anti-ableist agenda or strengths-based orientation, preferred by many autistic adults, is consistent with producing high-quality research. Both turn on the question of how different groups of autistic people are categorized and characterized, decisions which can affect how research participant groups are recruited and how research results are reported. At least some researchers and parents of autistic children argue that using what many autistic adults and allies characterize as anti-ableist language can result in researchers’ neglect of those populations most impacted by autism, including those with high support needs or significant intellectual disabilities. They have argued that “while scientific words may at times provoke feelings of social stigmatization in some people, the response should lie in addressing that stigma, not in eliminating fact-based, accurate descriptors in clinical, medical and scientific discourse,” especially given that there is evidence that there are a range of opinions within autism communities about preferred terminology for autism (Singer et al. 2022, 498; Flowers et al. 2023).

Those advocating this approach to language welcomed the U.S. Centers for Disease Control and Prevention’s (CDC) introduction, following the recommendation of a *Lancet* commission, of the category of “profound autism” as a way to ensure the counting and representation of children and adults who have significant learning, behavioural, and communication challenges in both research and policy domains (Lord et al. 2022; Hughes et al. 2023; Autism Science Foundation 2023). Others in the autism and autism research communities disagreed, arguing for example that other, more specific descriptors (e.g. “requiring 24-hour support” or measures of intellectual disability) are better suited to research needs (Natri et al. 2023). In response to the introduction of the profound autism category, Bottema-Beutel and colleagues (2023) pointed out that prior research programmes driven by an agenda not informed by the disability rights movement, for example one structured around the assumption that neurocognitive difference is by definition pathological, have produced flawed research. Others expressed concern that profound autism labels for individuals may also increase the risk of stigma, including the possibility that physical symptoms unrelated to autism may not be given appropriate diagnostic attention, and may encourage individuals interacting with those diagnosed with profound autism to discount their intelligence or latent communicative abilities (Woods, Williams, and Watts 2023; Kapp 2023). In response, those pushing for the use of this category argue that emphasizing the potential intelligence of individuals with high support needs may suggest that intelligence or communicative ability are what render people worthy of regard (Lutz 2024).

Although ethics is an important motivator for community-engaged practices, not all research described as “community-engaged” fully realizes ethical values like responsiveness to community needs and respect for communities. This can happen because of disproportionate attention to the interests of different groups within a community or because of practices that use community input instrumentally, in the absence of sustained engagement. Attention to the ethics of such studies—including the degree to which studies are designed to benefit communities and the degree to which communities maintain control of data—matters. As Mikesell, Bromley, and Khodyakov (2013) note, “CBPR is both a scientific method and an ethical practice” and it can be challenging to ascertain the appropriate balance between these two mandates. The same can be said of other forms of community engaged research. For this reason, and because of disagreements about the value, necessity, and optimal form of community engagement, it is important to hear from autism researchers in this moment when community engagement practices are slowly becoming more commonplace within autism research, particularly regarding what they understand to be the ethical benefits and potential ethical challenges of these practices.

While existing work (Hollin and Pearce 2019; Pickard et al. 2021) has examined autism researchers’ attitudes toward community engagement and their views of the benefits and drawbacks of such work, to our knowledge no one has yet asked investigators specifically about their views on the ethics of community-engaged research practices, meaning how such practices matter for the ethical implications of research design and the ethical conduct of research. As part of a project on stakeholder attitudes toward ethics and community engagement in autism research, we interviewed a set of autism researchers about these issues. We sought to identify the specific ways that researchers understand the potential for community engagement to change the practice of autism research in ethical terms, meaning in terms of the degree to which their research addresses issues like respect for persons, beneficence, and justice. We found that as community engagement becomes a more common practice in autism research, practitioners are developing additional perspectives on the value of such work, both for stakeholder communities and the research enterprise. More specifically, researchers emphasized how their interactions with autistic people and family members serving as advisory board members and co-investigators have changed their perspectives on several key issues. These include the specific value and utility of stakeholder input, the costs of participating in research for autistic people and their families, and following from that, specific principles of research design ethics. This paper describes the researchers’ perspectives in each of these areas, then elaborates on these findings.

## Methods

We recruited ten participants for this study following their participation in a survey of autism researchers that focused on research group characteristics and community engagement practices. All participants in that survey identified as independent autism researchers, meaning that they were a PI or similar level researcher able to conceptualize and conduct their own research without formal mentorship, which typically requires a PhD, MD, or other terminal degree. Participants in that longer survey were asked whether they would be willing to participate in a follow-up semi-structured interview and were contacted by a member of the research team if they were willing to participate. The ten participants interviewed in this study included three men and seven women. All participants were autism researchers, had published on autism-related topics and were active in the field at the time we interviewed them. We did not ask participants to specify their research area given concerns about identifying participants. We also did not ask whether they had received an autism diagnosis and none of the participants mentioned this in their interviews, though we are aware that some autism researchers have autism diagnoses themselves.

We received ethical approval from our Institutional Review Board for this project, and we obtained informed consent from all participants in the study. The study was supported by a grant from the U.S. National Science Foundation (NSF Award # 1737149) and no members of the research team had competing financial or non-financial interests. Interviews were conducted by a member of the research team via Zoom and took approximately one hour. Participants were compensated with a $25 gift card, the same level of compensation as stakeholder participants in an earlier phase of the study.

Interviews were professionally transcribed and cleaned by the team member who conducted the interviews. A subset of transcripts were then coded by three members of the research team using an inductive approach, meaning that the team members initially read the transcripts and identified recurring thematic categories that emerged from their reading rather than employing predetermined categories of analysis. The three team members then met to discuss their codes and create a consensus coding scheme. One team member then recoded the initial three transcripts as well as the remaining transcripts in NVivo (QSR International), a qualitative analysis software programme, meeting periodically with team members to discuss questions or issues with the coding scheme.

Because the interview participants had already agreed to take part in a survey on community engagement and had expressed interest in discussing the topic further, they were unlikely to be a representative sample of the autism research community as a whole, and we did not conduct our analysis with that assumption. Instead, we were interested in what a group of researchers with some awareness of community engagement and potentially direct experience of it thought about the ethical implications of these practices.

## Results

Researchers in our sample represented a group of participants that had already indicated some level of interest in producing knowledge about community engagement because of their prior participation in our survey. We found that this group, in their responses to our questions, pointed to specific examples of how engagement with community members, including autistic adults and children, service providers, and parents, shaped their research programmes in ways that mattered for the ethical implications of those programmes. This included changes in their thought processes, style of investigating problems, routine research practices, and research designs. Respondents explained that rather than simply informing researchers about alternative values related to autism or providing supplementary perspectives on research outcomes or dissemination of results, autistic or caregiver collaborators offered input that challenged and changed the researchers’ prior assumptions about autism, beliefs about what research participation entailed, and ideas about how to obtain the most valid results.

## The Value of Stakeholder Input

A significant feature of participants’ responses was the concrete examples they provided for how drawing on autistic expertise and direct experience enabled them to improve and refine their studies in ways that mattered to them both as scientists and as people. These included the more routinely cited example of using autistic peoples’ lived experience to inform study design and make research topics more relevant to community needs, in other words, to act as a supplement or enhancement to research. However, they also recognized that input that slowed the research process or challenged the process of inclusion and participation itself also had import because it improved the study’s ethics.

### Rethinking Research Design

A participant described one example where a study began as a project to receive feedback about assessment tools but with the input of autistic community members became a deeper interrogation of what defined the desirable outcomes that the tools were designed to measure.

The purpose of the study is to get feedback from the autistic community about these measurement tools and adapt a number of measurement tools. But in doing that, we also started with—“well, what construct should we even measure? What are meaningful to you? And that was really an important part of the study and gave us all new insight because things that they felt were important and what they thought would be an outcome for the service wasn’t always lined up well with what the providers thought would be an outcome of those services. (Participant 9)

Another said

We’re creating an autistic-centred questionnaire on “so you think you’re autistic.” And it’s a list of things that are just generally not reflected in our questionnaires or in the DSM, but are very, but feel like real autistic truth. (Participant 5)

Without explicitly invoking autistic collaborators or community feedback, two other researchers described how considering the social experience of autistic people caused them to think differently about the ethics of research practices or the goals of intervention-focused research. Both noted that the autism interventions that many children encounter may have the goal of increasing compliance. In some cases, this may be because educational goals for children include rules in service of safety, such as those related to traffic, hygiene, or household dangers. Nevertheless, encouraging compliance in children may lead to additional factors for researchers to consider when enrolling autistic adults in studies or assessing the safety of interventions:

So I think the basis of training towards compliance, for instance, and then when the people really do comply, they’re more vulnerable to abuse. […] When I review for [organizations that fund autism research] and when I see projects that come across that are about training people to comply, it makes me very concerned. (Participant 2)

Another explained that

… we put in multiple sort of circuit breaker points throughout the study where we sort of remind people, here’s what you’ve agreed to do, are you still okay to do that? Because I do think, especially kids with ASD who maybe are in schools and have learned by whatever methodology to be kind of compliant, if they’re told, well, this grown up told me to do this thing next and I’m supposed to do it next then. (Participant 3)

Participant 3 also noted that better research and a better-informed stakeholder community is a two-way street, where benefits run in both directions.

So there’s some studies that we’ve done over the years that we’ve sort of tried to create projects that build a better bridge between the autistic community and the scientific community so that the—so the science can be more informed by experience and experience can be more attuned to what’s going on kind of in the scientific world, right? So that doesn’t sort of feel so distant because I do think that there is sort of a—there’s a gap both ways. (Participant 3)

And Participant 4 noted how important it is to include autistic people in their research in some way, while also nodding to the effort required to answer competing imperatives, in this case training and mentoring students while also including autistic community members early in the research process.

And so I think that I need to be more mindful in my research about mentoring students appropriately while also getting input from a greater variety of autistic people about what could go on for this research. It’s new for me. I’m really learning. (Participant 4)

### Communication and Shared Meaning

Similarly, researchers explained how sustained interaction with autism community members helped them understand, and in some cases agree with, positions that seemed difficult to support, leading them to present studies differently. Becoming more attuned to how expert language can alienate underserved communities can also help researchers work more effectively with stakeholders.

For example, one researcher talked about how, in studies on complementary and integrative therapies,

… actually having to sit there and listen to parents discuss the choices that they make has been really good for me as a researcher because I think I was, as a healthcare provider, really judgy about it. (Participant 7)

Another commented that they had learned that when family members used the term “facilitated communication,” apparently referring to practices that many researchers do not consider to be supported by evidence, they were actually using it as a synonym for augmentative and alternative communication (AAC), which covers communicative supports generally recognized as effective. They explained that:

… sometimes if you just ask what it is that they’re doing, you find out that that’s not at all what they’re doing. They’re basically supporting somebody and learning how to use an augmentative device, but they’re not actually providing support that would look like facilitating communication. So sometimes people get so overreactive at the words that are used without understanding what it is that people are trying to communicate. (Participant 1)

Similarly, another commented that communities that are not accustomed to high degrees of access to educational support might not understand what the goals of a study were without better explanation, highlighting how community engagement can help researchers become better aware of the exclusionary aspects of technical language:

So it’s almost like we’re talking [in] a different language. I’ll say, “Oh […] the things we learn in this study can improve your child’s IEP," and I had someone with a teenager on the spectrum raise their hand and say to me “But what’s an IEP?" (Participant 3)

### Ethics and Practice

Participants also discussed examples of where community engagement about the process of inclusion and facilitating respect for persons, rather than the content of research, was critical to doing research in ways that they perceived as ethically appropriate. This was even true when input led to the temporary slowing of work or to discomfort on their part.

You have to be satisfied with having that internal sense of feeling like I’ve done this the right way and not maybe saying, “well, my autistic co-investigator gave me 25 vetoes when we were doing this together." Because and even if that’s the truth, those vetoes are actually really easy because the thought is always, “oh thank goodness you found that.” Because I’ve been doing this long enough that I know that it’s not a hard thing. It’s a real gift. But if I were to say 25 vetoes, then nobody is going to want to do that kind of partnered research because that sounds hard to them. They want to be able to just do what they want. But we’re so wrong all the time. (Participant 5)

Another researcher described a “powerful experience” that occurred during a meeting with autistic scholars and autism researchers:

Some of the autistic individuals felt like their voices weren’t being effectively heard. And I said in the context of the conversation, “I feel like I want to help—I want to help reach out to scholars who don’t know how to think about these things in an inclusive way. And one of the autistic individuals on the call said something to me that I’ll never forget. They said, “[Name], you’re not one of the people who knows how to think about these things.” And it took me a long time to kind of hear that, right, because my reaction was sort of defensive and I felt offended. […] I think any time that I or another scientist sort of sits on our laurels and believes we’ve sort of reached that pinnacle, that we sort of figured it out, I think that’s the first place we start to trip up. (Participant 3)

As Participant 3 recognized, being an autism researcher committed to community engagement did not guarantee that they were themselves experts on inclusive or collaborative research design or guaranteed to be attentive to ethical problems with research just because they had become better attuned to the potential for problems than some of their colleagues. The value of autistic collaborators was that they were continually available to identify issues that the researchers could not or did not identify on their own:

We also have mechanisms where we hire autistic individuals as part of our research team, or we have them work as part of a research team as a research intern. And that [is a] very, very important component because without that voice kind of constantly, we’re potentially researching things that aren’t relevant for that community, our community, or don’t actually make sense. And we clearly have found that that’s the case, that without their input, our research would not be as relevant as it should be. (Participant 9)

To be clear, not all researchers were unreservedly supportive of community priorities, nor did they suggest that stakeholder beliefs were always aligned with their own understanding of established information about autism or what they believed to be credible approaches to therapy. One mentioned how in theory a researcher might accept funding from a foundation that supported a therapy they considered discredited but keep their thoughts to themselves to maintain good relations with the funder. Another talked about the difficulty of managing the preferences of parents who were not comfortable accepting an autism label for their child. In other words, participants described at least some cases where becoming aware of stakeholder preferences might lead researchers to withhold opinions rather than engage in an open exchange of ideas.

Participant 5 noted that inclusion of autistic people in research design is not only about bringing their perspectives into the research but also can act as a protectant against researchers’ previously unexamined assumptions, challenging them to consider their research design more carefully at early stages.

I think that when autistic voices and perspectives are included from the very, very beginning … really, it serves more than one purpose at a time. I think the most important purpose is ethical. But close on its heels is that they really allow us as researchers or intervention developers or measure developers—they really save us from ourselves. And it’s a very cost-effective way to do research because you can fast forward like five or ten years by including autistic co-investigators kind of from the start. (Participant 5)

Put differently, community engagement from the outset of research reduced the likelihood of early, escalating missteps that would be far more challenging to correct once study aims were set.

## The Demands of Research Participation

A second way that participants described their ethical thinking as being influenced by community engagement (or at least awareness of stakeholders’ priorities and experiences) was in terms of their understanding of research participation as labour. This extended to their consideration of the effects of research on participants and their recognition of autistic collaborators’ work.

One researcher described problems with Institutional Review Board (IRB) capacities. They explained that IRBs were in some cases operating with so little understanding of contemporary autism research that it was difficult for those evaluating protocols to picture autistic participants completing study tasks.

I think often IRBs, even though they are the adjudicators of the ethics of research, they truly don’t know about the ethics of autism research today. And I’ve often had to acculturate IRBs—I and my colleagues have—to what’s involved. So meaning, they would say, “That is too demanding,” or “We can’t imagine someone with autism would do this or could do this task." (Participant 3)

Another researcher explained how seeing the costs to autistic collaborators in terms of the energy required of them to collaborate on research helped them think differently about the costs and benefits of participation in studies more generally. While IRBs are guided to consider the potential benefits to participants and the potential risks, considerations of participation as labour are more limited. Research may not present a physical or psychic risk, but it still may require that autistic research participants expend energy in different ways than would be true for neurotypical participants:

But sort of that cost of being an autistic researcher translated into a measure about the cost for the autistic participant to be in a brain imaging study and a phenotype study, for example, right. How long did you have to recover, right? So it’s an ethical consideration but it’s also for partners. But it’s also an ethical consideration for researchers all over. (Participant 5)

Another researcher explained that the IRB at their institution would inform them that their compensation rate for study participants was too high and potentially coercive, under the standard assumption that an excessive rate of compensation might be coercive, meaning encourage participants who would otherwise opt out to enrol. The researcher countered that adequate compensation was the only way to recognize that participants were expending time, energy, and thought on the study in the same way as other collaborators.

And we’d have to come back and say, “I don’t understand why that’s coercive. That’s kind of standard pay. That’s what you would pay these other populations. This is what you would pay providers. Why is a community member’s time not considered as valuable as a provider’s time?” (Participant 9)

Intangible recognition also matters. A participant who worked with autistic collaborators explained the importance of acknowledging and crediting their collaborators’ efforts (on the questionnaire described earlier): “From the beginning, I thought it was really important to make clear that the two autistic co-investigators are first off authors on that measure” (Participant 5).

Other researchers described ethical concerns related to investigating interventions that would not be widely affordable or accessible or researching aspects of autism that didn’t have the potential to yield benefits to the community. They did not invoke the labour of study participants in either of these examples, but both reservations relate to the idea that researchers have a responsibility to communities that provide the materials and data that supports the research enterprise.

Is this really going to help a lot of people? If you want to study, for instance, a drug, and nobody’s going to be able to afford it anyway, I’m like, “Well, I mean, should we be doing this or not?,” things like that. (Participant 2)

And so sometimes we think that developing new technology for autism identification is going to be a really good thing so we can identify kids earlier, but in fact, it could be a good thing only for some people and could actually exacerbate autism disparities. And so one of my projects right now is about how can we develop new technologies for autism that include all people. (Participant 7)

One researcher who did not recount community engagement in their work nonetheless suggested that participants might be voting with their feet when they chose to leave a study because the demands of participation might be intolerable. They explained that:

Another important value—it’s hard to know how to put this, and it’s what keeps me up at night. It’s how hard can we push people for research […] Obviously, we rely on autistic people to share their experience with us, but I feel we can abuse that privilege sometimes. (Participant 4)

## Responsibility to Participants and Research Design Principles

Researchers who discussed collaborating with autistic adults were not the only ones who responded to community concerns with changes to their study methods. Several participants described the importance of ensuring that children could be included in studies (a priority of their parents) and that inclusion in a study meant that everyone had an opportunity to receive the active treatment or intervention if they desired. Participant 10 explained:

And for us, we’ve really tried to think carefully about that everybody who says they want to participate in a treatment study gets the active treatment at some point if they so choose. So they’re not just doing the research to just sort of do it but they—the potential for benefit is there. (Participant 10)

They continued,

We try to have really broad inclusion criteria so that we include as many kids who have lots of complexity that kind of parallel the real world so that it isn’t this very narrow inclusion criteria where somehow all the hard kids are excluded and we only get this one slice of mild kids. These are pretty complex kids and families that we work with. And we want to do that intentionally. (Participant 10)

Another participant explained how families that wanted their children to participate in a study all hoped to have access to the treatment under investigation, even though in a randomized controlled trial, a control group would not receive an intervention. Addressing this issue as a matter of equity meant that they had to change the format of their studies and hire additional personnel to accurately interpret results. They explained:

But it’s very important for the community. They didn’t want a control group to not have an opportunity, or to be invited, to also receive the intervention. And that’s very understandable. (Participant 9)

They continued that to accommodate that desire, their team had moved to a study format involving “multiple baselines,” but with a large number of participants:

And so, all of [the study sites] would have a control period, and that would be staggered, and then they would all receive the intervention period. So that makes sense. And the community was very interested in that. But from a research team, then we had to go and find a whole new statistician who knew how to support us in data analysis in those kinds of methodologies. They are much more complicated. And we also have to, kind of, work with funders to, kind of, understand those new methodologies as well, because that’s not the standard classic design in a research proposal. (Participant 9)

Participant 9 explained how study designs that responded to community needs by providing broader access to experimental interventions are more difficult to execute, both because of scepticism on the part of funders and the greater complexity of these methods. Indeed, although Participant 9 explained that multiple baseline studies offered a way to accommodate community preferences, Participant 2 explicitly cited multiple baseline studies as ethically problematic, noting that groups needed to be

… finding big enough numbers to get real effect sizes as opposed to the smaller multiple baseline studies that inflate effect sizes and mislead people. So you get thousands of these small studies that allege that something works. (Participant 2)

## Discussion

Participants in this study offered examples of how they had learned and benefitted from engaging with autism stakeholder communities. They talked about how autistic peoples’ and their families’ ethical concerns helped them think about the risks of research participation and consent differently. They also discussed how stakeholders enabled them to understand barriers to communication that could otherwise undermine respect and distract from shared goals. Finally, they talked about how community engagement in a variety of guises changed their sense of research itself as an ethical and interpersonal undertaking.

Autistic children and adults and their family members participate in scientific research for a variety of reasons, but any engagement with the research process, whether as a participant, a collaborator, or an independent researcher, involves the expenditure of time and effort. Many of the researchers in our study, whether they described utilizing community engagement themselves or not, recognized this reality and tried to address it. This parallels arguments in bioethics that fear of coercion has led to inadequate compensation and thus potential exploitation of research participants (Largent and Lynch 2017). Participants in our study addressed these issues not only via compensation (as in Participant 9’s argument to their IRB that “standard pay” was applicable to research participants) but also by thinking about how to ensure that all participants in trials could have a chance to receive an experimental treatment or intervention, by recognizing that even research with minimal physical risk may be exhausting or require recovery, or by slowing the research process down in order to address concerns on the part of community members. Many of the researchers we spoke with were attuned to how any research project is in some sense a collaboration with participants. This helped guide some of their interactions with research participants and their sense of how research goals should align with the needs of autism stakeholder communities. In their relationships with autistic research collaborators, this could mean expressing gratitude and being explicit about their collaborators’ contributions or committing to co-authorship on publications.

Many of these changes take the form of negotiations around time. These include addressing parents’ desire for interventions that can address their children’s developmental trajectories and education,  responding to the desire on the part of some autistic people for meaningful inclusion in research, and on the part of researchers for results that can be reported to funders. Researchers acknowledged that sharing responsibility for research with stakeholders often meant shifting expectations about the pace of research, which parallels findings in other studies (Fraser-Barbour et al. 2025). Rethinking research design is one way to attempt to address the desire for research that is both responsive to community needs and potentially yields timely results. Designing larger, more powerful studies can increase the pace of research, but producing results more quickly can also contribute to potential harms at a moment when misleading reporting on effect size or level of certainty have been cited as growing ethical concerns. This is because of how gaps in health communication can leave out information about the “effect size of benefits and harms of health behaviours or interventions” (Brown 2023, 87).

Changes in study design can also come at a cost to researchers because they involve locating personnel who can analyse more complex data, educating funders about the validity of less conventional methods, and convincing colleagues that research results are reliable. As we saw with the participant who was dismissive of multiple baseline studies, choosing to work this way can mean encountering sceptical colleagues, many of whom are mindful of how poorly supported or unsupported interventions have harmed children with autism and their families in the past. For example, although Participant 9’s group had designed a study using a large group, all of whom would eventually have access to the intervention, and had hired a specialized statistician to address some of the potential shortcomings of studies of this type, they understood that funders might nonetheless worry that behavioural intervention studies not using a conventional format produce less reliable and trustworthy results. Similarly, Vivanti (2022) contrasts studies of efficacy, where interventions are administered to homogenous groups under tightly controlled conditions, and studies of effectiveness, meaning how well interventions work in real-world settings, implemented by personnel with less specialized training and delivered to more heterogenous populations. Effectiveness studies may address issues of equity in participant populations by including a more representative sample of participants as well as those with multiple diagnoses, but they can also introduce both confounding variables and bias, rendering their results less generalizable.

Nonetheless, several of our participants expressed their willingness to potentially sacrifice rapidity of output and professional regard in order to utilize community engagement practices and expressed gratitude to autistic collaborators for providing feedback that enabled them to avoid or remedy errors or explore avenues that they might otherwise have ignored. This is important to note because a major concern among bioethicists discussing the role of community input in autism research has been the possibility that uneven or disproportionate representation of different groups in community-engaged projects could bias input in favour of one stakeholder group andmight have a chilling effect on research. These groups could include, for example, parents who are committed to particular interventions not supported by evidence (e.g. Facilitated Communication) or autistic adults whose support needs are comparatively minor compared to those of others.

Our participants suggest that this may not be the most productive way to view the problem. Few of them raised the issue of over or under-representation of stakeholder groups, instead focusing on concrete and specific examples of how input from autistic individuals, service providers, and caregivers helped them think differently about *specific problems* in study conception and design. These were problems that researchers might otherwise have lacked the inclination, ability, or experience to raise or address themselves. In other words, autistic participants and family members were not determining the content of research programmes or directing programmes to orient research toward issues aligned with their own needs or interests, though in a true community engaged research project they *would* be selecting the research topic. Instead, they were helping researchers do better at their existing work on established research programmes, enabling more just and potentially more robust research.

This does not mean that concerns related to partial representation are not a consideration for autism researchers. As the disagreements detailed in our results about the relative risks and benefits of compliance training illustrate, some autism-related interventions can have both potential risks and potential benefits about which community members with different levels of support needs can have significant disagreements. The concern that the priorities of one stakeholder group might displace those of another is understandable. Part of the value of community engagement, our participants suggest, lies not in the power to determine research design, meaning to permit or veto any given topic, or to fully resolve contested issues, but in expanding the considerations researchers bring to their work. This includes ethical considerations related to harms, equitable distribution of benefits, accessibility, and participant autonomy.

The barriers to community-engaged research that our participants did describe were in line with concerns noted elsewhere in the literature, including problems making these methods legible to collaborators and funders, a difficulty that may make researchers reluctant to seek feedback on research priorities from stakeholders outside their professional community. Previous research has highlighted the need to train IRBs to handle community-engaged research protocols (Tamariz et al. 2015). It has also shown how difficult it can be to showcase community engaged methods in publications resulting from such work, when publications may not provide clear ways to identify research as community-engaged (Wright et al. 2014). Lack of infrastructure at the organizational level can also have a chilling effect. For example, the Autistic Researchers Committee of the International Society for Autism Research (INSAR) was formed in 2020 as a way to “advance the interests of autistic researchers within INSAR and advocate for adequate inclusion of this group across all INSAR programs” (International Society for Autism Research (International, n.d.), ¶1). This move led to the 2022 creation of the INSAR Community Collaborator Request (ICCR), a tool on the INSAR website to connect autism researchers with stakeholder collaborators, with the goal of improved “alignment between research outputs and the goals of the greater autism community, and consequently an increase in the overall quality and relevance of autism research” (Poulsen et al 2023, 2074). However, as one person familiar with the initiative explained, the site was hampered by lack of visibility and accessibility issues, leading to low participation.

As we noted in our description of our methods, these findings may be limited by the fact that the researchers interviewed had already agreed to both participate in a survey related to community engagement and autism research ethics and then offered to speak further with investigators about this topic. The group is therefore more likely to represent researchers who have already given considerable thought to community engagement and ethics, as indicated by their willingness to spend time participating in our project. They could therefore overrepresent the degree to which autism researchers as a whole actively utilize community engagement practices. It is also worth noting that by focusing on Ph.D. or M.D.-level researchers able to supervise projects, we could be excluding examples of citizen science or informal research conducted by members of different stakeholder groups. However, we think that our participants’ insights as those with greater institutional authority and experience with community engagement can be of value to researchers with less experience in this area or those who are interested in community engagement, but are unclear about how it might apply to their own work.

## Conclusion

There is growing awareness of and interest in community-engaged research within the autism research community. In addition to the availability of useful guidelines for research of this type, more studies are demonstrating the utility of community engaged research for helping to prioritize community needs and ensure respect for stakeholder communities. Although prior work has analysed researchers’ views on community engagement in general, no previous study has asked them specifically about the ethical dimensions of these practices, though community engagement is both a research practice and an ethical orientation (Mikesell, Bromley, and Khodyakov 2013). The present study demonstrates that autism researchers and community stakeholders both see the potential for community engagement to address numerous ethical challenges, including those related to the labour involved in research participation and ensuring that the potential benefits and hazards of research participation are shared equitably across autism stakeholder groups.

## Data Availability

Not applicable.
